# The evaluation of Bcftools mpileup and GATK HaplotypeCaller for variant calling in non-human species

**DOI:** 10.1038/s41598-022-15563-2

**Published:** 2022-07-05

**Authors:** Messaoud Lefouili, Kiwoong Nam

**Affiliations:** DGIMI, Univ Montpellier, INRAE, Montpellier, France

**Keywords:** Bioinformatics, Software, Genetic variation

## Abstract

Identification of genetic variations is a central part of population and quantitative genomics studies based on high-throughput sequencing data. Even though popular variant callers such as Bcftools mpileup and GATK HaplotypeCaller were developed nearly 10 years ago, their performance is still largely unknown for non-human species. Here, we showed by benchmark analyses with a simulated insect population that Bcftools mpileup performs better than GATK HaplotypeCaller in terms of recovery rate and accuracy regardless of mapping software. The vast majority of false positives were observed from repeats, especially for GATK HaplotypeCaller. Variant scores calculated by GATK did not clearly distinguish true positives from false positives in the vast majority of cases, implying that hard-filtering with GATK could be challenging. These results suggest that Bcftools mpileup may be the first choice for non-human studies and that variants within repeats might have to be excluded for downstream analyses.

## Introduction

The identification of genetic variation constitutes a central part of population genomics and quantitative genomics studies. Single nucleotide variations (SNVs) are particularly informative for inferences of selective sweeps, demographic history, and phylogeny, as well as for quantitative analyses such as genome-wide association studies and quantitative trait loci. These approaches rely on mathematical models with the assumption that natural selection is not directly acting on the analyzed genetic variations. Therefore, unbiased variant calling from a large quantity of sequencing data is indispensable. The improvement in high-throughput sequencing techniques has enabled the generation of large-scale genomic SNV datasets from multiple samples.

**Figure 1:**
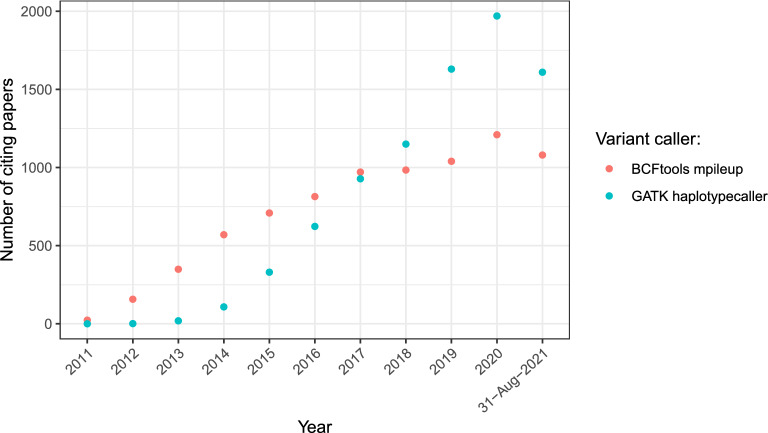
The number of papers citing 'mpileup' or 'HaplotypeCaller' (source: Google Scholar).

Several popular software packages have been developed for variant calling. In particular, Samtools mpileup (now Bcftools mpileup) was previously the most widely used variant caller^1^. However, it has now been overtaken by GATK HaplotypeCaller^2^ (Fig. 1), partly because GATK HaplotypeCaller performs much faster than Bcftools mpileup for large numbers of samples. GATK HaplotypeCaller is widely regarded as the best option for variant calling; for example, one paper^3^ states, ‘The current gold standard for variant-calling pipelines is the Genome Analysis Toolkit (GATK) Best Practices Workflow pipeline using HaplotypeCaller, which is considered to have the highest accuracy for single nucleotide polymorphisms (SNPs) and small insertions and deletions.’ The majority of benchmark studies have concluded that GATK HaplotypeCaller outperforms Bcftools mpileup (Table 1).

**Table 1:** Studies comparing the performance of variant calling between GATK and Bcftools.

Year	Authors	Species	Better variant caller between Bcftools (Samtools) and GATK	Citation
2013	O'Rawe et al.	Human	Inconclusive	^15^
2013	Liu et al.	Human	GATK	^16^
2013	Yu and Sun	Human	GATK UnifiedGenotyper	^17^
2014	Cheng et al.	Human	Inconclusive	^18^
2014	Pirooznia et al.	Human	GATK HaplotypeCaller	^19^
2014	Yi et al.	Human	GATK HaplotypeCaller	^20^
2015	Cornish and Guda	Human	GATK UnifiedGenotyper > GATK HaplotypeCaller > Samtools mpileup	^21^
2015	Highnam et al.	Human	GATK HaplotypeCaller	^22^
2015	Hwang et al.	Human	Bcftools mpileup	^23^
2016	Laurie et al.	Human	GATK HaplotypeCaller	^24^
2017	Sandmann et al.	Human	GATK HaplotypeCaller	^25^
2019	Kumaran et al.	Human	Bcftools mpileup	^26^
2019	Wu et al.	Tomato	GATK HaplotypeCaller	^27^
2020	Schilbert et al.	Arabidopsis	GATK HaplotypeCaller	^28^
2021	Alosaimi et al.	Human	Bcftools mpileup	^4^

We question, however, whether GATK HaplotypeCaller should be the first choice of a variant caller because there are three issues yet to be answered. Firstly, the performance of variant callers in non-human species is relatively poorly understood as most benchmark studies are based on humans. Secondly, the studies did not evaluate the performance from SNVs segregating in populations. In principle, there are two sources of SNVs identified from resequencing data. The first source is SNVs for which the genotype differs among individuals in a population (P); in P, SNVs are polymorphic. The second source is the differences between the most recent common ancestor of the analyzed individuals and the reference genome sequences (D). In D, SNVs are not polymorphic because all individuals have the same genotypes. Since only P is informative for population genomics or quantitative analyses, the usefulness of variant callers needs to be compared from P. If a single read contains genetic variations from both P and D, D might interfere with variant calling from P. The extent of the interference may depend upon the level of divergence between the reference genomes and the most recent common ancestor (*d*). Thirdly, benchmark studies based on simulation have rarely been performed on a biologically realistic population, for which the process of variant calling is presumably affected by allele frequencies (however, see Alosaimi et al.^4^).

Filtering potential false-positive SNVs is an essential step in variant calling. In Bcftools mpileup, filtering can be performed readily from variant calling score, which is a phred-scaled probability of false variant calling. GATK HaplotypeCaller provides two ways of filtering. The first option is hard filtering, which discards SNVs if variant scores are lower or higher than certain thresholds, which are typically determined by humans. These variant scores include QD (the variant quality score normalized by read-depth), FS (the phred-scaled probability of strand bias), SQR (strand bias calculated by symmetric odds ratio test), MQ (the root mean square of mapping quality of reads mapped to a nucleotide), MQRankSum (the normalized difference in mapping qualities between reference and alternative alleles), and ReadPosRankSum (the normalized difference in position between reference and alternative alleles within the reads)^5^. The second option is VQSR (Variant Quality Score Recalibration). In this method, the threshold of variant scores is determined by machine learning from truth and training resources generated from a reference SNV data set. Such truth and training resources do not exist in the vast majority of non-human species.

In this study, we compared the performance of variant calling from P with varying *d* in an insect population between GATK HaplotypeCaller and Bcftools mpileup using benchmark analyses. Since a reference SNV data set is not available for most non-human species, we performed benchmark analysis from a simulated insect population, which mimics *Drosophila melanogaster*.

## Results

### Variant calling together with bowtie2

**Figure 2:**
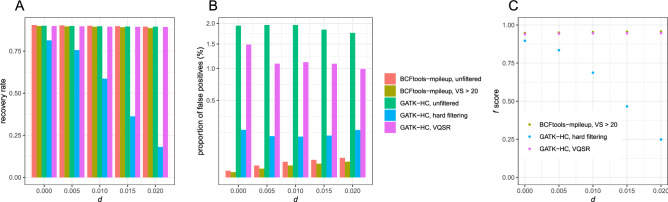
Variant calling performance using bowtie2 (**A**) The recovery rate, (**B**) the proportion of false positives, and (**C**) *f* scores of SNV datasets generated by Bcftools or GATK.

First, we performed mapping of high-coverage reads (40 × per individual) using bowtie2. The mapping rate ranged between 75.96 and 77.05%, depending upon *d*. The recovery rates were comparable between Bcftools mpileup (89.53–90.49%) and GATK HaplotypeCaller (89.48–90.01%) (Fig. 2A). The recovery rates were negatively correlated with *d* both for Bcftools mpileup (ρ = − 0.999, p = 5.115 × 10^–5^; Spearman's correlation test) and GATK HaplotypeCaller (ρ = − 1.000, p = 1.128 × 10^–5^), while the range of the recovery rate is less than 1%.

Bcftools mpileup had lower proportions of false positives (0.00373–0.0321%) than GATK HaplotypeCaller (1.759–1.959%) by 54–521 times. Bcftools-mpileup had a positive correlation between the proportion of false positives and *d* (ρ = 1.000, p = 0.0004293), while this trend was not observed from GATK HaplotypeCaller (ρ = − 0.858, p = 0.06269). As a consequence, the ratio of the proportion of false positives from Bcftools mpileup to GATK HaplotypeCaller was positively correlated with *d* (ρ = 1.000, p = 1.53 × 10^–5^).

When *d* equals zero, the majority of false positives were observed from repeats (78.91% and 85.24% of total false positives for Bcftools mpileup and GATK HaplotypeCaller, respectively) in line with a previous study^6^. GATK HaplotypeCaller generated a higher proportion of false positives at repeats among total false positives than Bcftools mpileup by 54% (p = 0.03585; two-tailed fisher’s exact test). GATK HaplotypeCaller generated a particularly high number of false positives at LTR transposons and LINE transposable elements (Table 2). In addition, GATK HaplotypeCaller generated disproportionally abundant false positives at simple repeats, low complexities, and satellites.

**Table 2:** The numbers of false positives according to different repeats generated by GATK HaplotypeCaller and Bcf mpileup, and the ratio of false positives from GATK HaplotypeCaller to Bcf mpileup.

Repeat type		GATK-HaplotypeCaller	BCFtools mpileup	Ratio
Non-repetitive sequence		11,441	31	369
Transposable elements	LTR	44,145	55	803
	DNA	4748	50	95
	LINE	13,053	9	1450
	Rolling-circle	1167	0	NA
Total transposable elements		74,554	145	514
Simple repeat, low complexity, Satellite		2637	2	1319
Other repetitive elements		344	2	172

Then, we tested the effect of filtering. When SNVs with variant calling scores lower than 20 were discarded from the SNV data of Bcftools mpileup, the recovery rate ranged between 88.67 and 89.65% (Fig. 2B), which is lower than the unfiltered data set just by 0.59–0.86%. The recovery rates were negatively correlated with *d* (ρ = − 0.988, p = 0.001551). The proportion of false positives ranged between 0.0023 to 0.0286%, which is lower than the unfiltered data set by 35–42%, and it was positively correlated with *d* (ρ = 1.000, p = 3.854 × 10^–5^).

**Figure 3:**
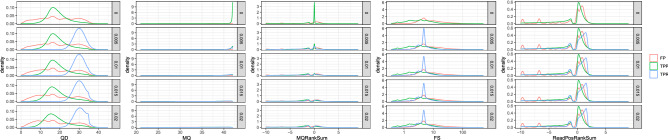
Variant scores calculated by GATK. The density plots of five scores of true positive and false positive SNVs. TPP, TPR, and FP are true positives from P, true positives from D, and false positives, respectively. Please note that the heights do not show relative abundance among TPP, TPR, and FP.

**Figure 4:**
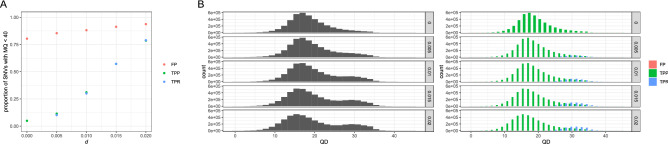
MQ and QD scores in GATK. (**A**) The proportion of TPP, TPR, and FP with MQ < 40 according to *d*. (**B**) The histograms of QD scores (left) from all SNVs and (right) from TPP, TPR, FP, separately.

For GATK-HaplotypeCaller, no variant score discriminated clearly between true positives and false positives (Fig. 3). The exception was MQ scores with low *d*. For example, when *d* = 0, 80.39% of false positives had MQ scores lower than 40, while only 4.929% of true positives had MQ scores lower than 40 (Fig. 4A). On the other hand, when *d* = 0.02, 93.74% of false positives and 78.56% of true positives from P had MQ scores < 40. This result implies that MQ < 40 filterings will cause a severe reduction in the recovery rate by discarding 78.56% of true positives. Interestingly, a bimodal distribution of QD scores was observed except for a case with *d* = 0 (Fig. 4B, left). Each mode in this bimodal distribution appears to represent SNVs from P and D (Fig. 4B, right).

We performed hard-filtering according to the suggestions in the Technical Document of GATK^5^, which includes MQ < 40. GATK-HaplotypeCaller had much lower recovery rates than Bcftools mpileup (18.09–81.36%). The recovery rate rapidly decreased as *d* increased (ρ = − 0.984, p = 0.002369) (Fig. 2B). Furthermore, the proportion of false positives was 0.14–0.1910%, which was much higher than that of Bcftools mpileup (0.0023–0.0286%).

We also used VQSR filtering, which is based on machine learning with a training dataset to choose optimal criteria on variant scores, to perform filtering from the SNV data of GATK-HaplotypeCaller. For this purpose, we generated different sets of Illumina reads from the same 20 individuals with diploid genomes. The recovery rate was comparable (89.28–89.84%) to that of Bcftools mpileup, and it was negatively correlated with *d* (ρ = − 0.986, p = 0.002014). However, the proportion of false positives (0.9906–1.486%) was much higher than that of Bcftools mpileup, even higher than GATK hard filtering.

*f* scores calculated from P were highest at the filtered datasets of Bcftools mpileup (0.9468–0.9566), intermediate at GATK VQSR (0.9398–0.9462), and lowest at GATK hard filtering (0.2485–0.8965) (Fig. 2C). Bcftools and GATK VQSR generated largely invariable *f* scores across *d* whereas *f* scores from GATK hard filtering rapidly decreased as *d* increased.

### Variant calling together with bwa-mem

**Figure 5:**
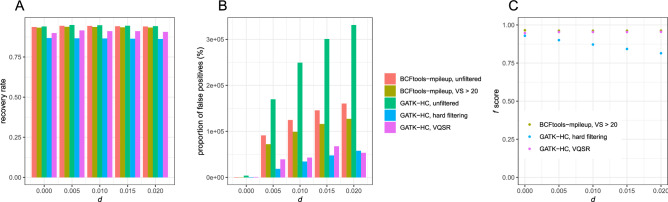
Variant calling performance using bwa-mem (**A**) The recovery rate, (**B**) the proportion of false positives, and (**C**) *f* scores of SNV datasets generated by Bcftools or GATK.

We compared performance between Bcftools and GATK HaplotypeCaller using a different aligner, bwa-mem with high coverage data (40 × for each sample). The recovery rates were comparable between Bcftools mpileup (93.71–94.42%) and GATK HaplotypeCaller (94.02–94.96%) (Fig. 5A). After filtering, Bcftools mpileup had the highest recovery rate (93.29–93.81%), followed by GATK VQSR (89.90–91.57%), and then by GATK hard filtering (86.25–86.80%).

Bcftools mpileup had lower proportions of false positives (0.018–2.50%) than GATK HaplotypeCaller (0.091–5.061%) (Fig. 5B). After filtering, the proportion of false positives was comparable between GATK hard filtering (0.202–1.27%) and GATK VQSR (0.204–1.22%), and Bcftools mpileup had a highest proportion of the proportion of false positives (1.94–2.30%) except for *d* = 0 (0.0101%).

After filtering, Bcftools mpileup had a slightly higher *f* score (0.9632–0.9674) than GATK VQSR (0.9623–0.9652), but substantially higher than GATK hard filtering (0.8146–0.9292) (Fig. 5C). GATK hard filtering showed a severe reduction in *f* scores as *d* increased.

### Low coverage data

**Figure 6:**
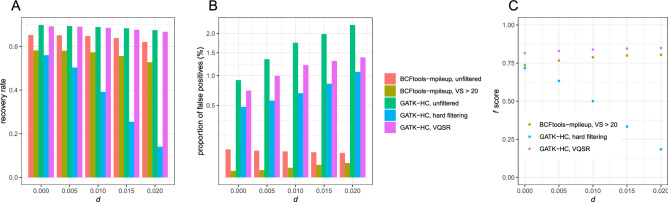
Variant calling performance from low coverage data (**A**) The recovery rate, (**B**) the proportion of false positives, and (**C**) *f* scores of SNV datasets generated by Bcftools or GATK.

Then, we analyzed low coverage data (5 × coverage per sample) with bowtie2. GATK Haplotycaller had higher recovery rates (67.49–69.85%) than Bcftools mpileup (62.07–65.21%) in unfiltered SNV datasets (Fig. 6A). After filterings, the recovery rate was highest in GATK VQSR (66.75–69.22%), intermediate in Bcftools mpileup (52.78–58.18%), and lowest in GATK hard filtering (14.03–56.00%).

GATK Haplotycaller had higher proportions of false positives (0.915–2.244%) than Bcftools mpileup (0.058–0.076%) in unfiltered SNV datasets (Fig. 6B). After filterings, the proportion of false positives was highest in GATK VQSR (0.726–1.39%), intermediate in GATK hard filtering (0.481–1.076%), and lowest in Bcftools mpileup (0.004–0.02%).

After filtering, *f* score was highest in GATK VQSR (0.8156–0.8482), intermediate in BCF mpileup (0.7356–0.8039), and lowest in GATK hard filtering (0.1840–0.7167) (Fig. 6C). In particular,  *f* score decreased rapidly as *d* increased in GATK hard filtering.

## Discussion

The identification of genetic variations is a critical step in studies based on high-throughput sequencing data. Both false positive and false negative SNVs might mislead population and quantitative genomics analyses. Thus, a variant caller needs to be chosen carefully although the performance of variant callers is largely unknown in non-human species, especially in invertebrates. In this study, we compared the performance of the two most popular variant callers, Bcftools mpileup and GATK HaplotypeCaller, in a simulated insect population.

### Performance of BCFtools and GATK HaplotypeCaller

In the dataset based on the mapping by bowtie2, when filtering was applied to the SNV data from Bcftools mpileup by discarding SNVs with variant calling scores less than 20, the proportion of false positives was reduced by 35–42% while the recovery rates were largely unchanged. When hard filtering was applied to the SNV data generated by GATK HaplotypeCaller, both the proportion of false positives and the recovery rates were substantially reduced. When VQSR filtering was used, the recovery rate was comparable with the filtering of Bcftools mpileup, while VQSR filtering had much higher proportions of false positives than Bcftools mpileup.

The effect of *d* is slight in all cases with a single exception of GATK-hard filtering, which had severely decreased the recovery rates when *d* was high. For example, when *d* was increased from 0 to 0.02, the recovery rate was reduced from 0.8948 to 0.1809. *f* statistics show that the best result can be obtained from the filtered SNV dataset from Bcftools mpileup. We also found that repeats are the main source of false positives, especially for GATK HaplotypeCaller. Taken together, Bctfools mpileup performs better than GATK HaplotypeCaller in terms of recovery rate and the correctness at both raw and filtered SNV datasets.

When bwa-mem was used for mapping, the same trend was observed with the exception that GATK had lower proportions of false positives (0.018–1.271%) than Bcftools mpileup (0.0101–2.304%) after filtering. It should be noted, however, that bowtie2 generated much lower proportions of false positives (0.0023–0.021%) than bwa-mem when Bcftools mpileup was used for variant calling.

When the coverage was low (5 ×) and bowtie2 was used for mapping, GATK VQSR and Bcftools mpileup had higher recovery rates, lower proportions of false positives, and higher *f* scores than GATK hard filtering after filtering, implying that GATK hard filtering had the lowest performance.

Taken together, these results show that Bcftools mpileup outperforms GATK HaplotypeCaller after hard filtering, regardless of the software used for mapping or the coverage of reads, especially when *d* was high. GATK VQSR appears to outperform Bcftools mpileup only when coverage is low according to the* f* score.

### Potential causes of the differential performance

The different performance between Bcftools mpileup and GATK HaplotypeCaller can be due to the way of handling alignments generated by a mapper. Bcftools mpileup uses alignments of a mapper as it is. Bcftools mpileup uses mapping scores to evaluate variant calling in a way that the variant calling score of an SNV is not allowed to be higher than the mapping score. The mapping score is a Phred-scaled probability of unique mapping, by definition. If a read is mapped to a repeat, the mapping score is likely to be low because the read could be also mapped to other similar repeats. Thus, Bcftools mpileup is able to handle potential false positives at repeats by discarding ambiguous mappings using the mapping score. GATK HaplotypeCaller adjusts an alignment by taking account of reads that are mapped to overlapping sequences. This correction is based on the assumption that a read was mapped to the right region in a reference genome with a possibility of local misalignment. If a read is mapped to an incorrect region in a reference genome, then a false-positive cannot be fixed by the adjustment of an alignment. Since mis-mappings are presumably more abundant at repeats, we believe that the higher number of false positives from GATK HaplotypeCaller is, in a large part, due to the mismappings at repeats. It should be noted that false positives can also be enriched at multi-copy genes, duplicated chromosome segments, and even DNA motifs for protein binding for the same reason.

The relatively low performance of GATK hard filtering is potentially due to the fact that most scores do not discriminate between false positives and true positives (Fig. 3). The exception is the MQ score with low *d*. When *d* = 0, filtering by MQ < 40 effectively removed false positives while the recovery rate was largely unchanged. However, the difference in MQ scores between true positives and false positives was decreased as *d* increased. As a consequence, hard filtering according to MQ scores appears to reduce the recovery rate significantly.

The distribution of QD scores can be informative to determine whether SNV filtering according to MQ scores. The distribution of QD scores was different between P and D (Fig. 4) and a bimodal distribution was observed when *d* > 0. The distribution of QD scores appears to be determined by the spectrum of alternative allele frequencies. SNVs from D are expected to have higher alternative allele frequencies than that from P. Therefore, if a significant proportion of SNVs was generated from D, a bimodal distribution of QD can be generated. In other words, a bimodal distribution of QD scores can be a signature that a significant proportion of SNVs was generated by D*.* In this case, filtering according to QD score is likely to reduce the recovery rate from P severely, and VQSR is the only reliabme method of filtering. If a unimodal distribution of QD score was observed, on the other hand, filtering according to QD score will effectively reduce false positives while the recovery rate is largely unchanged. On the official GATK homepage, a bimodal distribution of QD scores is explained as, 'This is because hom-var samples contribute twice as many reads supporting the variant than do het variants'^5^. Our results show that this explanation is may mislead because the distribution of QD scores reflects the frequency of alternative alleles in a population, instead of hetero or homozygosity of each sample.

Our result may appear to contradict previous papers showing that GATK HaplotypeCaller performs better for human data than Samtools/Bcftools mpileup. A majority of these studies assessed the performance by the comparison of an SNV dataset from a variant caller and another dataset ascertained by previous experiments. As mentioned by Heng Li, this comparison ‘would only give us an estimate of the relative accuracy––if two pipelines are affected by the same artifact that a third pipeline does not have, then the third pipeline will appear worse even though it is in fact better. In addition, comparative studies usually measure the accuracy with summary statistics such as the fraction of calls present in dbSNP or the transition-to-transversion ratio. They do not tell us the wrong sites’^6^. Indeed, obtaining correct SNVs positions across the *whole* genome is almost impossible from empirical data, thus evaluation of variant callers can be consequently biased. A list of *whole* true positive SNVs can be obtained only from a simulated population for unbiased comparison. However, it should be noted that any simulated population can be systematically different from a real population. Thus, we might be yet to reach a conclusion on which variant caller performs better between GATK HaplotypeCaller and Bcftools mpileup even in human studies.

### The choice of a variant caller

Here, we propose the following for variant calling in non-human species. First, Bcftools mpileup should be considered for variant calling over GATK HaplotypeCaller in non-human studies because Bcftools mpileup may generate a lower number of false positives and because SNV filtering according to variant calling scores may reduce effectively false positives while recovery rate remains largely unchanged. Second, SNVs within repeats should be excluded for downstream analysis, especially for GATK HaplotypeCaller. Third, when GATK HaplotypeCaller is used, we strongly advise using reference genome sequences that are not highly diverged from an analyzed population. Fourth, if the QD score from GATK shows a unimodal distribution, hard-filtering according to MQ scores can effectively reduce false positives while the recovery rate is largely unchanged; Fifth, if the QD score from GATK shows a bimodal distribution, VQSR filtering should be used. If a training dataset for VQSR is not available, Bcftools mpileup can be used as an alternative. Sixth, if a researcher wants to minimize the proportion of false-positive SNVs, we recommend using bowtie2 for mapping, rather than bwa-mem.

## Methods

### Simulation for data generation

A forward simulation was performed to generate a population of *Drosophila melanogaster* using Slim2^7^. The population size was 10^6,8^. The length of simulated DNA is 110,338,342 bp, which is the total length of autosomes in BDGP Release 6 (https://hgdownload.soe.ucsc.edu/goldenPath/dm6/bigZips/). The recombination rate and the mutation rate were 2.8 × 10^–9^ and 2.0 × 10^–8^, respectively^9,10^. For computation feasibility, the population size, mutation rate, and recombination were rescaled to 10^3^, 2.8 × 10^–6^, and 2.0 × 10^–5^, respectively. This re-scaling essentially generates the same result of simulation since the population-scaled mutation rate and recombination rate remain unchanged. The simulation was performed during 3,000 generations (equivalent to 3 × 10^6^ generations without rescaling) until we collected the genotypes of random 40 diploid individuals. The first alleles of these 40 diploid individuals were mapped to the autosomal reference genome of BDGP Release 6 with a randomly chosen alternative nucleotide sequence, followed by the generation of 20 diploid genomes by combining two haploid genomes.

Simulated paired-end Illumina reads were generated from each diploid genome using neat-genreads v3.0^11^ with 20 × coverage, which corresponds to 40 × coverage for a haploid reference genome. The read length was 150 bp. The average insert length was 300 bp and the standard deviation was 30 bp. The reference genomes were generated from the autosomal sequences of BDGP Release 6 by the introduction of new mutations with varying phylogenetic distances, *d* (0.5%, 1%, 1.5%, and 2%), using simuG v1.0.0^12^. The autosomal sequences of BDGP Release 6 were also used for the reference genome sequence. The genomic coordinates of repeat sequences within BDGP Release 6 were obtained from the table browser of UCSC Genome browser (https://genome.ucsc.edu/cgi-bin/hgTables).

### Mapping

The reads were mapped against these genome references using bowtie2 v2.3.5.1^13^ with the default setting of end-to-end mapping or bwa-mem2 v2.2. The resulting bam files were sorted according to the coordinates using samtools-1.9^1^. A read-group was assigned to each bam file using AddOrReplaceReadGroups function from the Picard tool v2.9.4^14^. The resulting bam files were indexed using samtools-1.9.

### Variant calling and filtering

We performed haplotype calling for each bam file using the HaplotypeCaller function at GATK v4.2.0.0^2^. The resulting gvcf files were merged into a single gvcf file. Variant calling was performed from this gvcf file using GenotypeGVCFs function. Then, SNVs were collected from the resulting vcf file. Hard Filtering was performed with the following criterion: QD < 2, FS > 60, MQ < 40, MQRankSum < − 12.5, ReadPosRankSum < − 8. For VQSR filtering, we generated two sets of 40 × simulated reads from the 20 diploid genomes. Then, variant calling was performed using HaplotypeCaller and GenotypeGVCFs at GATK. The resulting two vcf files with the information of true positives and false positives were used to recalibrate variant scores, and VQSR filtering was performed from the recalibrated variant scores.

Variant calling was also performed using Bcftools v1.9 with ‘bcftools mpileup -f REFERENCE LIST_OF_BAM | bcftools call -mv -Oz -o VCFFILE’ command, where REFERENCE, LIST_OF_BAM, and VCFFILE are the name of the reference genome sequence, the list of bam files, and the resulting vcf file name, respectively. Then, only SNVs were collected. Filtering was performed by discarding SNVs in which the variant calling score at QUAL field is lower than 20.

### Evaluation of performance

True positives and false negatives of the SNVs introduced by slim2 v2.0 were counted using custom java scripts. The recovery rate was calculated by TP/(TP + FN), where TP and FN are the numbers of true positives and false positives, respectively.

If an SNV was not found from the list of mutations generated by slim or SimuG, this SNV was identified as a false positive. The proportion of false positives was calculated by the number of false positives divided by the total number of SNVs in a vcf file. *f* scores were calculated by (2 × Recovery rate × Proportion of false positive)/(Recovery rate + Proportion of false positive).

## Data Availability

All scripts used in this study are available at https://github.com/kiwoong-nam/Variant-caller-comparison. The simulated reads are available on request.
